# Identification of BC005512 as a DNA Damage Responsive Murine Endogenous Retrovirus of GLN Family Involved in Cell Growth Regulation

**DOI:** 10.1371/journal.pone.0035010

**Published:** 2012-04-13

**Authors:** Yuanfeng Wu, Xinming Qi, Likun Gong, Guozhen Xing, Min Chen, Lingling Miao, Jun Yao, Takayoshi Suzuki, Chie Furihata, Yang Luan, Jin Ren

**Affiliations:** 1 Center for Drug Safety Evaluation and Research, State Key Laboratory of New Drug Research, Shanghai Institute of Materia Medica, Chinese Academy of Sciences, Shanghai, China; 2 Division of Cellular and Gene Therapy Products, National Institute of Health Sciences, Tokyo, Japan; 3 Department of Chemistry and Biological Science, School of Science and Engineering, Aoyama Gakuin University, Kanagawa, Japan; East Carolina University, United States of America

## Abstract

Genotoxicity assessment is of great significance in drug safety evaluation, and microarray is a useful tool widely used to identify genotoxic stress responsive genes. In the present work, by using oligonucleotide microarray in an *in vivo* model, we identified an unknown gene BC005512 (abbreviated as BC, official full name: cDNA sequence BC005512), whose expression in mouse liver was specifically induced by seven well-known genotoxins (GTXs), but not by non-genotoxins (NGTXs). Bioinformatics revealed that BC was a member of the GLN family of murine endogenous retrovirus (ERV). However, the relationship to genotoxicity and the cellular function of GLN are largely unknown. Using NIH/3T3 cells as an *in vitro* model system and quantitative real-time PCR, BC expression was specifically induced by another seven GTXs, covering diverse genotoxicity mechanisms. Additionally, dose-response and linear regression analysis showed that expression level of BC in NIH/3T3 cells strongly correlated with DNA damage, measured using the alkaline comet assay,. While in p53 deficient L5178Y cells, GTXs could not induce BC expression. Further functional studies using RNA interference revealed that down-regulation of BC expression induced G1/S phase arrest, inhibited cell proliferation and thus suppressed cell growth in NIH/3T3 cells. Together, our results provide the first evidence that BC005512, a member from GLN family of murine ERV, was responsive to DNA damage and involved in cell growth regulation. These findings could be of great value in genotoxicity predictions and contribute to a deeper understanding of GLN biological functions.

## Introduction

Genotoxicity assessment plays an important role in both toxicity screening during early drug discovery and regulatory drug safety evaluation in the preclinical stage [1]. Although a great number of genotoxicity assays have been developed, there is still a requirement for tests with both high specificity and sensitivity [2]. The use of microarray technology in toxicology, known as toxicogenomics, can potentially identify novel genotoxicity biomarkers and provide mechanistic insights into the mode of action of genotoxic compounds [3], [4], [5], [6], [7], [8]. We identified an unknown gene BC005512 (official full name: cDNA sequence BC005512), whose expression was specifically induced by genotoxins (GTXs) but not by non-genotoxins (NGTXs) in an *in vivo* microarray study. Elevated expression of BC005512 has been reported previously in thymocytes of Parp-2 deficient mice [9], suggesting that it is relevant to DNA damage. Further analysis of this gene uncovered that it is a member of the GLN family of murine endogenous retrovirus (ERV).

ERV sequences, most probably originating from infections of germ-line cells by ancient exogenous retroviruses during evolution [10], account for approximately 8% of the human genome [11] and 10% of the mouse genome [12]. ERVs were once thought to be junk DNA, but a number of studies have shown that some have important physiological roles [13], [14], [15] or are implicated in certain diseases [16], [17]. Several studies have reported elevated expression of ERV-related sequences in hepatocarcinogen treated rodents [18], [19].

The GLN family, designated due to an unusual primer-binding site sequence corresponding to tRNA^Gln^, is one of a number of murine ERV families. It was first identified over two decades ago [20], but remains little-studied [21], [22]. The relationship between GLN and genotoxic stress and the biological function of GLN family members are largely unknown. Here we report that BC005512, a member of the GLN family of murine ERV, was responsive to DNA damage and involved in regulation of cell growth.

## Results

### 1. Selection of specific and sensitive genotoxic stress responsive genes using microarray

**Figure 1:**
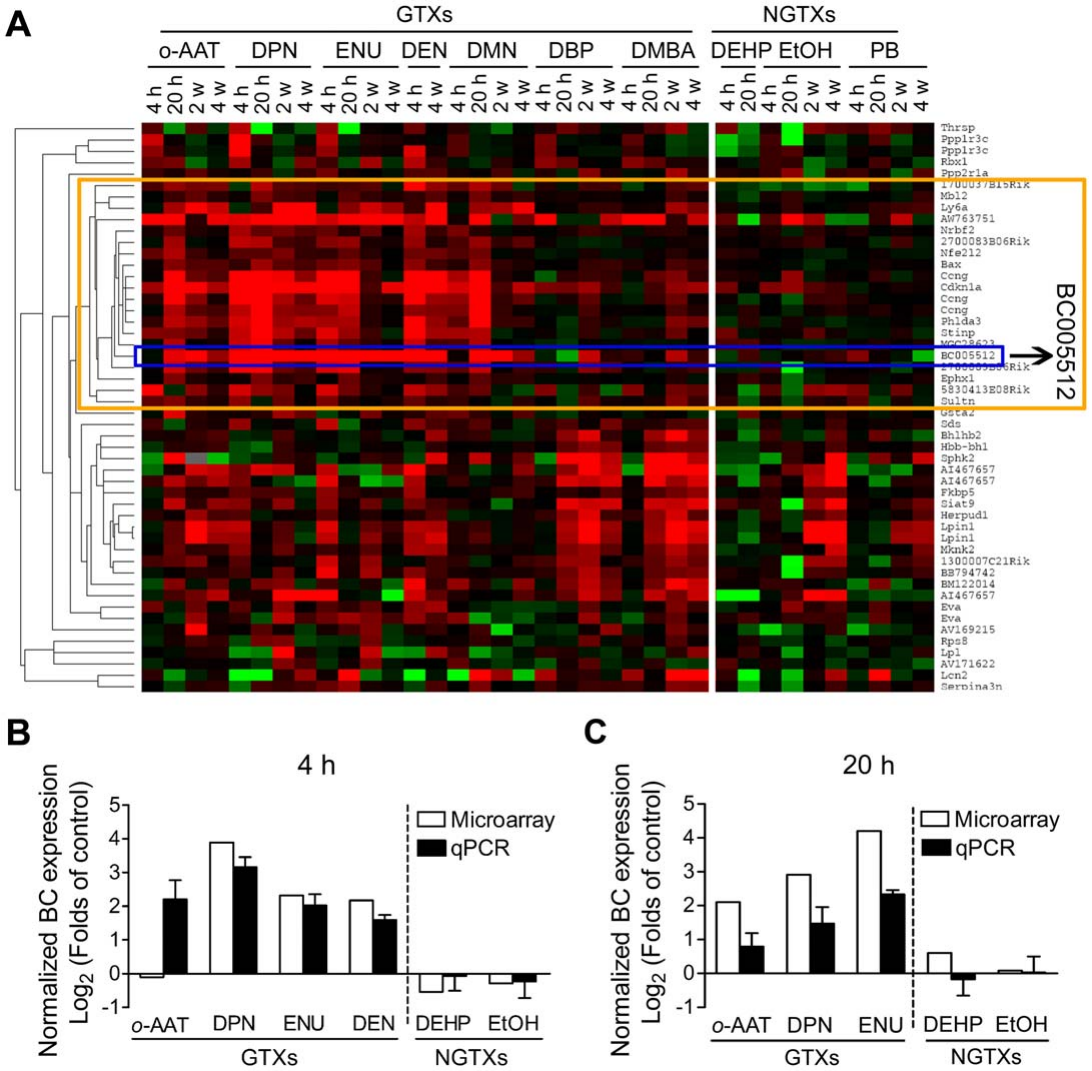
Selection of sensitive and specific genotoxic stress responsive genes. (**A**) Hierarchical clustering of top 50 scored up-regulated genes shown in gene symbol. Red and green indicate up-regulation and down-regulation, respectively. The orange box represents genes whose expression could distinguish GTXs from NGTXs. The blue box represents the gene with the highest score, BC005512. (**B** and **C**) Microarray and quantitative PCR (qPCR) data showing BC expression levels in livers of mice dosed with indicated chemicals at 4 h or 20 h after administration. Microarray data represented pooled samples from 4 animals per group. Quantitative PCR data were mean ± s.d. (n = 4).

Microarray is a powerful way of examining genomic scale gene expression changes. To identify specific and sensitive genotoxic stress inducible genes, we carried out an *in vivo* microarray study specifically investigating liver tissue in B6C3F1 mice administered with seven well-characterized genotoxins (GTXs) and three non-genotoxins (NGTXs). Compounds with all negative data in regulatory genotoxicity assays (including Ames test, *in vitro* chromosome aberration test, mouse lymphoma assay and *in vivo* micronucleus test) were chosen as non-genotoxins. The dosage used for GTXs was selected based on data from *in vivo* transgenic mouse mutation assays, where significantly higher mutant frequencies were observed in liver tissue. The mutant frequency was determined as described previously [23]. While the dosage used for NGTXs was 1/2 LD_50_ (Table 1). To study both early and late or sustained genotoxic stress responses, time points at 4 h, 20 h, 2 weeks and 4 weeks after treatment were chosen. To select genotoxic stress responsive genes, we adopted a self-defined weight scoring approach. Candidate genes were scored based on their specificity, sensitivity (including average ratio, positive condition, positive chemical and reverse change), statistical *P* value, basal expression level, and coefficient of variation (CV). A total score, considering all the above parameters, was finally calculated (Table 2). Further analysis of the top ranked 50 genes by hierarchical clustering showed clear gene sets, whose expression could distinguish GTXs from NGTXs (Fig. 1A). These included some well-known DNA damage inducible genes e.g. p21^WAF1/Cip1^ [24] and ccng1 [25]. The highest scoring gene was an unknown gene BC005512 (identified by probe set 1426936_at, Gene symbol: BC005512, official full name: cDNA sequence BC005512). Its expression was specifically induced by GTXs, but not by NGTXs, which was further confirmed by quantitative real-time PCR (Fig. 1B and 1C).

**Table 1:** Model compounds selected in the *in vivo* microarray study.

Compounds	Abbr^1^	CAS^2^ No.	Dosage (mg/kg)	Classification
Dimethylnitrosamine	DMN [23]	62-75-9	5	Genotoxins
Diethylnitrosamine	DEN [46]	55-18-5	80	
Ethyl nitrosourea	ENU [47]	759-73-9	150	
Dipropylnitrosamine	DPN [48]	621-64-7	250	
*o*-Aminoazotoluene	AAT [49]	97-56-3	300	
Dibenzo[*a,l*]pyrene	DBP [50]	191-30-0	6	
7,12-Dimethylbenz[*a*]anthracene	DMBA [48]	57-97-6	20	
Ethanol	EtOH	64-17-5	1000	Non-genotoxins
Phenobalbital sodium	PB	57-30-7	30	
Diethylhexylphthalate	DEHP	117-81-7	2000	

1 Abbr: Abbreviation;

2 CAS: Chemical Abstracts Service.

**Table 2:** Weight score for genotoxic stress responsive gene selection in the *in vivo* microarray study (liver, B6C3F1).

Systematic	Specificity		Ave ratio		Positive condition		Positive chemical		*P* value		Basal		Reverse change		CV (%)		TOTAL SCORE	Common
	V^1^	S^2^	V	S	V	S	V	S	V	S	V	S	V	S	V	S		
1426936_at	1.00	5	10.62	5	11.5	5	6	5	0.14	5	0.92	0	0.0	2	64	2	**32.0**	BC005512
1417185_at	1.00	5	3.98	2	14.5	5	6	5	0.07	5	2.35	3	0.0	2	27	4	**31.5**	Ly6a
1433691_at	1.00	5	3.31	2	5.0	2	5	4	0.21	4	2.64	4	2.0	5	24	4	**28.5**	Ppp1r3c
1449002_at	1.00	5	4.40	2	12.0	5	6	5	0.33	2	1.80	1	0.0	2	26	4	**27.5**	Phlda3
1421040_a_at	1.00	5	2.49	2	5.5	2	5	4	0.18	4	2.63	4	0.0	2	33	4	**27.0**	Gsta2
1424638_at	0.83	3	24.58	5	15.0	5	7	5	0.19	4	1.51	0	0.0	2	70	2	**27.0**	Cdkn1a
1450016_at	1.00	5	3.26	2	11.0	5	5	4	0.35	1	2.13	2	0.0	2	12	5	**26.5**	Ccng
1450017_at	1.00	5	4.55	2	11.0	5	5	4	0.29	2	1.79	1	0.0	2	23	4	**26.5**	Ccng
1420827_a_at	0.92	4	7.88	4	11.0	5	5	4	0.33	2	1.75	1	0.0	2	43	3	**26.0**	Ccng
1416578_at	1.00	5	2.88	2	5.0	2	5	4	0.17	4	1.92	1	0.0	2	35	4	**25.5**	Rbx1
1424744_at	1.00	5	2.42	0	6.5	3	6	5	0.46	0	3.01	5	2.0	5	33	4	**25.0**	Sds
1425631_at	1.00	5	3.72	2	4.0	1	4	2	0.15	5	2.09	2	4.0	5	38	3	**25.0**	Ppp1r3c
1416125_at	1.00	5	2.49	2	5.0	2	4	2	0.12	5	2.28	3	0.0	2	71	2	**24.5**	Fkbp5
1419874_x_at	0.90	4	5.77	3	9.0	4	5	4	0.15	5	1.37	0	−1.0	1	107	0	**24.5**	AI467657
1427422_at	0.83	3	3.15	2	10.0	5	6	5	0.27	3	1.99	1	1.0	3	46	3	**24.5**	BM122014
1442026_at	0.83	3	3.70	2	10.0	5	5	4	0.11	5	1.62	0	0.0	2	50	3	**24.5**	AI467657
1416926_at	0.86	3	3.37	2	12.0	5	6	5	0.31	2	2.25	2	0.0	2	24	4	**24.0**	Stinp
1448265_x_at	1.00	5	2.55	2	7.5	3	6	5	0.36	1	2.02	2	−1.0	1	52	3	**24.0**	Eva
1455892_x_at	0.91	4	2.52	2	10.5	5	5	4	0.48	0	2.92	5	1.0	3	80	2	**24.0**	BB794742
1418787_at	1.00	5	1.89	0	5.0	2	5	4	0.31	2	3.16	5	0.0	2	13	4	**23.5**	Mbl2

1 “V” represents values.

2 “S” represents score. Only the top 20 genes are shown. A full list is attached in Table S3.

Detailed scoring rules are described in the supporting information. “Pink cells” represent up-regulation and “blue cells” represent down-regulation.

**Specificity** = (number of total pink cells in GTXs)/(number of total pink cells in GTXs and NGTXs); **Ave ratio** = average of ratios of all pink cells in GTXs; **Positive condition** = number of total pink cells in GTXs. Since DEN was duplicated, each pink cell was considered 0.5; **Positive chemical** = number of GTXs with at least one pink cell; ***P*** **value** was calculated by *t* test of signal intensity between GTXs and NGTXs in GeneSpring software; **Basal** represents basal expression level, equals to log_10_ value of signal intensity of control animals; **Reverse change** reflects opposite change of gene expression in different treatment groups. Reverse change = number of blue cells in NGTXs - number of blue cells in GTXs; **CV%** = 100×SD/MEAN% based on the signal intensity of all control animals. **Total score** = Score of 2× Specificity + Ave ratio + Positive condition + Positive chemical + *P* value + 0.5× Basal + 0.5× Reverse change + 0.5× CV%.

### 2. BC005512 is a member of the GLN family of murine endogenous retrovirus

**Figure 2:**
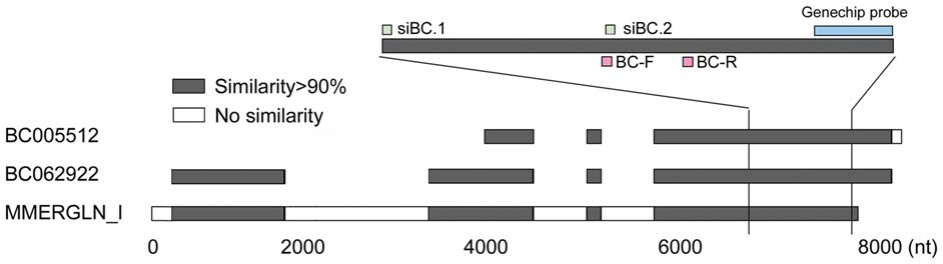
BC005512 is a member of the GLN family of murine endogenous retrovirus. Sequence alignment between BC005512, BC062922 and MMERGLN_I. Locations of the genechip probe, quantitative PCR primers (BC-F and BC-R) and BC siRNAs are shown.

To characterize BC005512 in the mouse database, we performed bioinformatics analysis. **A** nucleotide BLAST search using the genechip probe sequence (Fig. S1) as the query identified two cDNAs (GenBank accession no. BC005512 and BC062922). Analysis of both sequences using ORF finder (http://www.ncbi.nlm.nih.gov/gorf/gorf.html) and protein BLAST revealed that the putative proteins encoded by these sequences have great similarity to retrovirus related proteins encoded by *env* genes of mouse ERV (data not shown) [10]. To determine the relationship of BC005512 with mouse ERVs, we analyzed BC005512 and BC062922 sequences using an online CENSOR program (http://www.girinst.org/censor/index.php), which screens query sequences for interspersed repeats [26]. The sequence analysis classified them into ERV-Class I, and revealed strong similarities to MMERGLN_I, a sequence submitted as one copy of GLN family in Repbase (Fig. 2). These findings suggested that the sequence identified in the above microarray study, i.e. BC005512 (abbreviated as BC in the following text), was a member of the GLN family of murine ERV.

### 3. Expression of BC was specifically induced by genotoxins in NIH/3T3 cells

**Figure 3:**
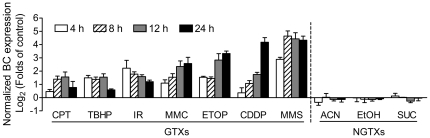
Expression of BC was specifically induced by GTXs in NIH/3T3 cells. Data from quantitative PCR showing transcriptional expression of BC in NIH/3T3 cells treated with genotoxic or non-genotoxic chemicals for indicated time. Data were mean ± s.d. of three independent experiments.

The microarray study showed that BC was induced by genotoxic stress *in vivo*. To further characterize the responsiveness of BC to GTXs *in vitro*, we tested another seven GTXs with different genotoxic mechanisms and two NGTXs (Table 3). For each GTX, 24 h IC_50_ was determined and used to study the effects of treatment on BC expression. NIH/3T3, a well-characterized mouse embryonic fibroblast cell line, was used as the *in vitro* model system. In accordance with results obtained *in vivo*, all seven GTXs up-regulated expression of BC to varying degrees, while all three NGTXs tested had no obvious effects on BC expression in NIH/3T3 cells (Fig. 3). Similar results were also obtained in a mouse hepatoma cell line, Hepa 1–6 cells (Fig. S2).

**Table 3:** Model compounds selected in the *in vitro* BC induction study.

Compounds	Abbr.	CAS No.	Primary modes of action	Concentration		Classification
				NIH/3T3	L5178Y	
Camptothecin	CPT	7689-03-4	Topoisomerase I poison [51]	25 µM	2.87 µM	Genotoxins
*tert*-Butyl hydroperoxide	TBHP	75-91-2	Reactive oxygen species [52]	50 µM	——	
γ-ray	IR	——	Ionizing radiation	8 Gy	10 Gy	
Mitomycin C	MMC	50-07-7	Bi-functional cross-link alkylating agent [53]	30 µM	6 µM	
Etoposide	ETOP	33419-42-0	Topoisomerase II poison [54]	50 µM	1.7 µM	
Cisplatin	CDDP	15663-27-1	Crosslink agent [55]	12.5 µM	——	
Methyl methanesulfonate	MMS	66-27-3	Mono-functional alkylating agent [27]	0.5 mM	——	
Acetonitrile	ACN	75-05-8		10 mM	10 mM	Non-genotoxins
Ethanol	EtOH	64-17-5		10 mM	10 mM	
Sucrose	SUC	57-50-1		10 mM	10 mM	

### 4. Induced expression level of BC correlated with DNA damage in NIH/3T3 cells

**Figure 4:**
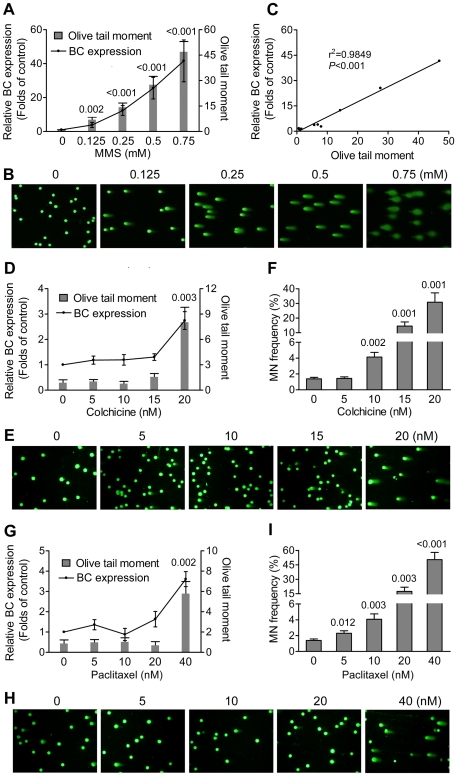
Induced expression level of BC correlated with DNA damage in NIH/3T3 cells. (**A, B, D, E, G and H**) Comparison between expression level of BC and DNA damage in NIH/3T3 cells exposed to MMS (A, B) for 8 h, or to colchicine (D, E) or paclitaxel (G, H) for 24 h. DNA damage was measured by olive tail moment (tail length × percentage of DNA in tail) in an alkaline comet assay (representative figures are shown in B, E and H). Data were mean±s.d. of three independent experiments. (**C**) Linear regression analysis between expression level of BC and DNA damage, reflected by olive tail moment. Each dot represents the mean of data shown in (A), (D) and (G). (**F** and **I**) Micronucleus frequency in NIH/3T3 cells exposed to colchicine or paclitaxel for 24 h. Data were mean ± s.d. of three independent experiments. Values shown on top of bars are *P* values *vs* control.

Inducing DNA damage is one of many genotoxicity mechanisms. To further study the relationship between BC expression and DNA damage, we compared transcriptional expression level of BC and the extent of DNA damage using methyl methanesulfonate (MMS) as a DNA-reactive model compound [27]. MMS was chosen as it gave the strongest response in the *in vitro* BC induction assay (Fig. 3) and has been used extensively as a DNA damaging model agent. MMS modifies both guanine (to 7-methylguanine) and adenine (to 3-methlyladenine) causing base mispairing and replication blocks, respectively [27]. DNA damage was indicated by olive tail moment in the alkaline comet assay [28]. As shown in Fig. 4A and 4B, MMS induced a concentration-dependent increase in both BC expression and DNA damage.

In addition to DNA-reactive chemicals, aneugens that directly target spindles but not DNA during chromosome segregation were another class of GTXs. To determine whether BC was responsive to aneuploidy, we examined the effects of two aneugens, colchicine [29] and paclitaxel [30], on BC expression. Chromosome abnormality was determined using the micronucleus test [31]. The highest concentration of paclitaxel or colchicine was limited to 48 h IC_50_. As expected, both colchicine and paclitaxel induced a dose-dependent increase in micronucleus formation (Fig. 4F and 4I) but not in DNA damage except at very high concentrations, possibly due to nonspecific effects under cytotoxic conditions (bar graph in Fig. 4D and 4G, 4E and 4H). In accordance with MMS treatment, quantitative PCR analysis showed that expression level of BC was in parallel with DNA damage i.e., BC induction only occurred when DNA damage was observed, regardless of whether aneuploidy was induced (line graph in Fig. 4D and 4G). Furthermore, the linear regression analysis of data obtained from MMS, colchicine and paclitaxel revealed a strong correlation between expression level of BC and the extent of DNA damage (Fig. 4C).

### 5. Expression of BC could not be induced by GTXs and did not correlate with DNA damage in L5178Y cells

**Figure 5:**
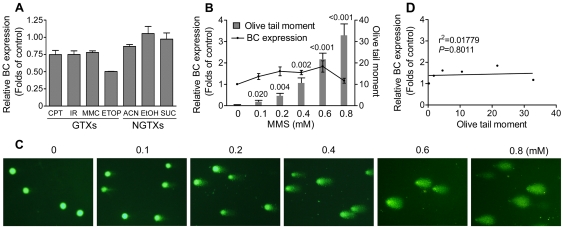
Expression of BC could not be induced by GTXs and did not correlate with DNA damage in L5178Y cells. (**A**) Quantitative PCR data showing transcriptional expression of BC in L5178Y cells treated with indicated chemicals for 4 h. Data were mean ± s.d. of three independent experiments. (**B** and **C**) Comparison between expression level of BC and DNA damage in L5178Y cells exposed to MMS for 8 h. DNA damage was measured by olive tail moment (tail length × percentage of DNA in tail) in an alkaline comet assay (representative figures are shown in C). Data were mean ± s.d. of three independent experiments. (**D**) Linear regression analysis between expression level of BC and DNA damage, reflected by olive tail moment. Each dot represents the mean of data shown in (B). Values shown on top of bars are *P* values *vs* control.

In addition to NIH/3T3 cells, L5178Y, a widely used mouse lymphoma cell line in *in vitro* genotoxicity assays was also adopted to investigate the effects of GTXs on BC expression and the relationship between BC expression and DNA damage. For each GTX, 24 h IC_50_ was determined and used in L5178Y cells. Surprisingly, GTXs that substantially induce BC expression in NIH/3T3 cells did not show similar effects in L5178Y cells (Fig. 5A and Fig. S3). Concordantly, expression level of BC did not correlate with DNA damage induced by MMS (Fig. 5B, 5C and 5D). Together, these results suggested that expression of BC could not be induced by GTXs and did not correlate with DNA damage in L5178Y cells.

### 6. Down-regulating BC expression suppressed cell growth in several mouse cell lines

DNA damage triggers a variety of biological responses including the transcriptional activation of genes regulating DNA repair, cell cycle checkpoint and cell death [32]. The observation that BC was responsive to DNA damage raised the question whether BC was involved in these processes.

**Figure 6:**
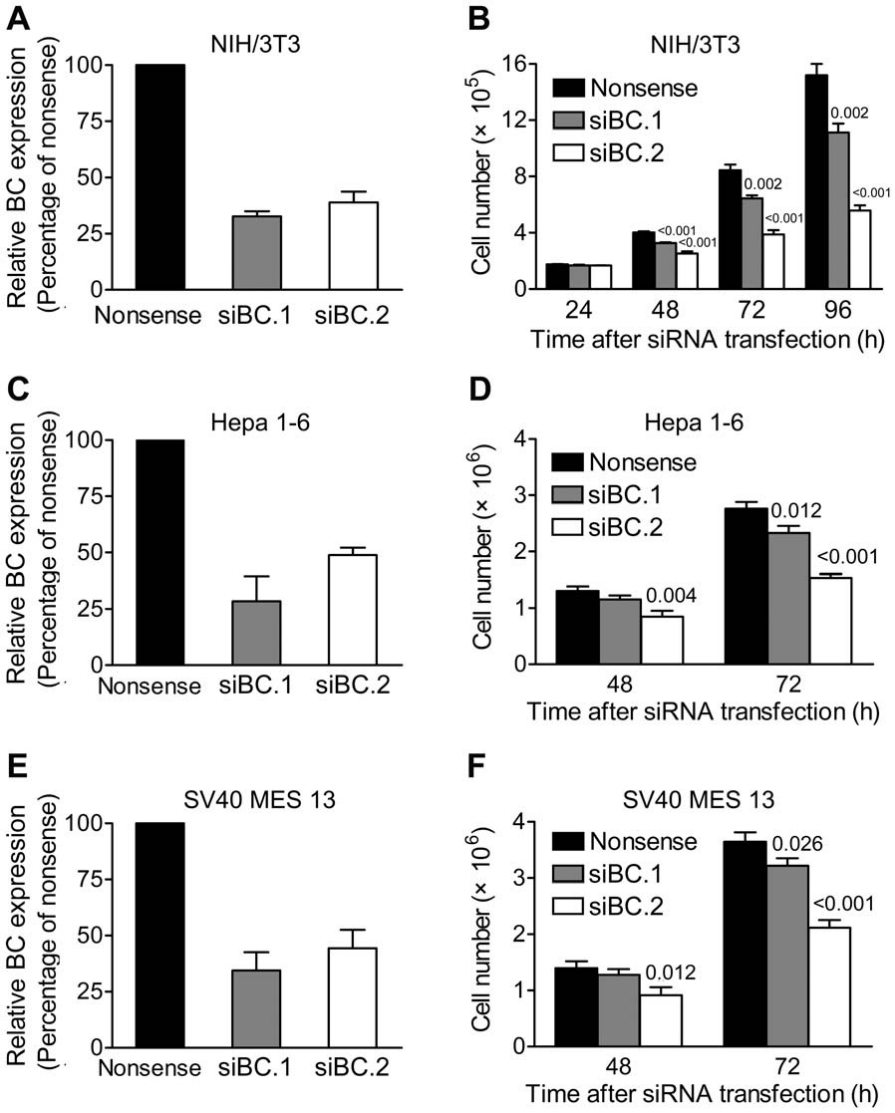
Down-regulating BC expression suppressed cell growth in several mouse cell lines. (**A, C** and **E**) Quantitative PCR results showing knock-down efficiency of BC siRNAs in NIH/3T3, Hepa 1–6 or SV40 MES 13 cells at 48 h after siRNA transfection. Data were mean ± s.d. of at least three independent experiments. (**B, D** and **F**) Cell numbers of NIH/3T3, Hepa 1–6 and SV40 MES 13 cells at indicated times after siRNA transfection. Data were mean ± s.d. of at least three independent experiments performed in triplicate. Values shown on top of bars are *P* values *vs* nonsense.

We first examined the effects of knocking-down BC expression on cell growth by RNA interference. Two BC siRNAs were designed; one nonsense siRNA and two corresponding scrambled siRNAs were used as negative controls. Quantitative PCR showed that at 48 h after siRNA transfection, the level of BC mRNA was markedly reduced in cells transfected with BC siRNAs (siBC.1 and siBC.2) compared with cells transfected with nonsense (Fig. 6A) or scrambled siRNAs (Fig. S4A). Due to the lack of appropriate antibody against BC, to confirm the knock-down efficiency of BC siRNAs at the protein level, we generated a myc-tagged BC clone. Following co-transfection of the clone and BC siRNA into NIH/3T3 cells, the protein expression level of BC was monitored using a myc-tag antibody. Meanwhile, we introduced two point mutations into the siBC.2 target region to generate a mutant clone. By co-transfecting this mutant clone and siBC.2 into NIH/3T3 cells, we further examined the specificity of siBC.2. As shown in Fig. S5, siBC.2 decreased the wild-type BC, but not the mutant BC protein levels, indicating that siBC.2 worked specifically and effectively at the protein level.

Cell growth analysis of NIH/3T3 cells transfected with BC siRNAs showed that the total cell number was markedly reduced compared with cells transfected with nonsense (Fig. 6B) or scrambled siRNAs (Fig. S4B). Similar results were also obtained in a mouse hepatoma cell line Hepa 1–6 (Fig. 6C and 6D), and a mouse glomerular mesangial cell line SV40 MES 13 (Fig. 6E and 6F). These results suggested that knock-down of BC expression suppressed cell growth in several mouse cell lines.

### 7. Knock-down of BC induced G1/S phase arrest and inhibited cell proliferation in NIH/3T3 cells

**Figure 7:**
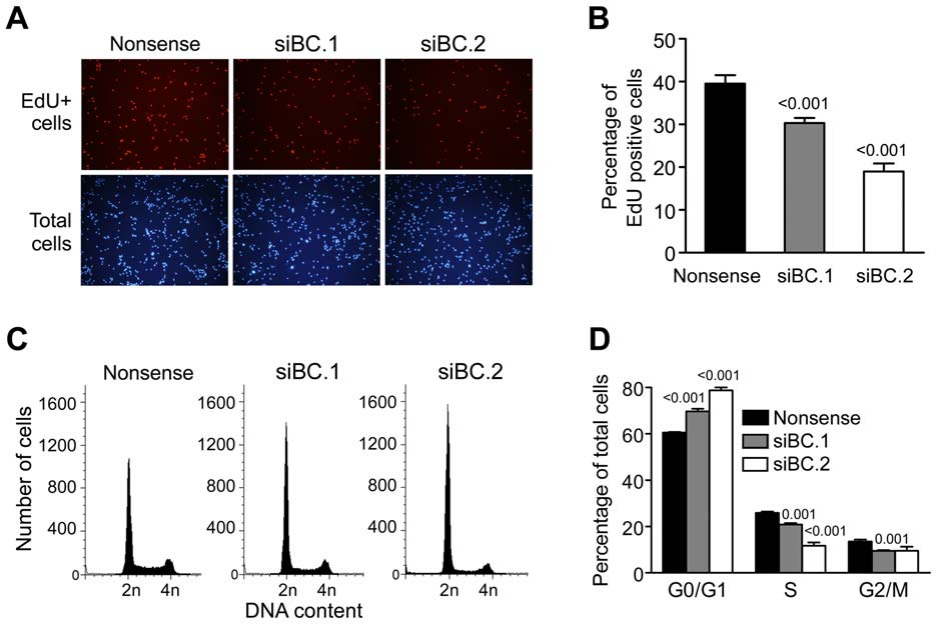
Knock-down of BC induced G1/S phase arrest and thus inhibited cell proliferation in NIH/3T3 cells. (**A**) Representative fluorescent images of EdU incorporation assay. Blue nuclei represent total cells visualized by UV excitation, while red nuclei represent EdU-positive cells visualized by green light excitation. (**B**) Quantitative analysis of EdU-positive cells (shown in A). A total of >4000 cells were counted for each group. Data were mean ± s.d. of three independent experiments. (**C**) Flow cytometric analysis of cell cycle in NIH/3T3 cells transfected with nonsense or BC siRNAs at 48 h after siRNA transfection. 2n = cells in G0/G1 phase, and 4n = cells in G2/M phase. (**D**) Quantitative analysis of cell cycle phase distribution (shown in C). Data were mean ± s.d. of at least three independent experiments performed in triplicate. Values shown on top of bars are *P* values *vs* nonsense.

To investigate whether BC affected cell growth due to its effect on cell proliferation or cell survival, we performed an EdU incorporation assay, which is similar to BrdU incorporation assay. As shown in Fig. 7A and 7B, fewer EdU-positive cells were found in BC siRNAs-transfected cells (30.3%±1.2% for siBC.1, and 19.0%±1.9% for siBC.2) than in nonsense siRNA-transfected cells (39.5%±2.0%), indicating that knock-down of BC suppressed NIH/3T3 cells proliferation.

To elucidate how knock-down of BC can suppress cell proliferation, we examined the effects of knocking-down BC on cell cycle. At 48 h after transfection, the percentage of cells in G0/G1 phase was substantially increased (60.5±0.3 for nonsense siRNA, 69.7±1.0 for siBC.1 and 78.7±1.3 for siBC.2) with a concomitant reduction of cells in S phase (25.9±0.5 for nonsense siRNA, 20.8±0.7 for siBC.1 and 11.7±1.4 for siBC.2, Fig. 7C, 7D and Fig. S4C).

To determine whether down-regulation of BC expression affected cell death, we examined apoptosis in NIH/3T3 cells by flow cytometry using Annexin V and propidium iodide double staining. Knock-down of BC did not induce apoptosis at 48 h after siRNA transfection (Fig. S6A). Consistently, no obvious sub-G1 peak was observed (Fig. 7C), indicating that down-regulation of BC did not induce apoptosis directly in NIH/3T3 cells. Nevertheless, the obvious apoptosis observed at 96 h after siRNA transfection (Fig. S6B) might be caused by cell cycle arrest, occurring at 48 h after transfection.

Taken together, these results suggested that down-regulation of BC induced G1/S arrest, and thus inhibited cell proliferation in NIH/3T3 cells.

## Discussion

In this work, we have shown that BC005512, a member of the GLN family of murine ERV, was responsive to DNA damage. This conclusion was supported by several observations. Multiple well-characterized GTXs with a diversity of genotoxicity mechanisms induced elevated expression of BC both *in vivo* (mouse liver) and *in vitro* (NIH/3T3 cells). More specifically, a strong correlation was found between expression level of BC and the extent of DNA damage. Besides the above findings, we provide the first evidence that BC was involved in cell growth regulation, suggesting that BC was biologically relevant to the DNA damage response.

Recent studies have reported elevated expression of ERVs in many different types of human cancers or tumor cell lines [17]. UVB irradiation was reported to induce transcriptional activation of ERV sequences in human epidermal keratinocytes [33]. However, the relationship between GLN and DNA damage was not clear. This is the first report that demonstrates the involvement of a GLN family member in genotoxic stress, particularly in the DNA damage response. This quantitative DNA damage-responsive property of BC could be potentially useful in genotoxicity prediction.

In addition to BC, we identified another ERV with a high score (1455892_x_at, BB794742, mouse endogenous murine leukemia virus mRNA), whose expression was induced by GTXs (Table 2). A great number of human ERVs have been reported to have perfect p53 binding sites that not only regulate adjacent gene expression, but may also give ERVs the advantage of exiting the host cell for their own survival under stress conditions [14]. As discussed later, we also found that the long terminal repeat (LTR) of GLN has a putative p53 binding site. Combined with our findings, it would be of great interest to study the relationship between ERVs and stress responses, and determine whether ERVs have intrinsic advantages in becoming potential genotoxicity biomarkers.

In the microarray study, BC was initially identified using self-defined weight scoring. In our scoring system, well-known DNA damage responsive genes such as p21 [24] and ccng1 [25] were also assigned high scores, indicating that this method worked effectively in identifying genotoxic stress inducible genes. Although the basal expression level was low, BC had priority over other candidate genes in its high specificity and sensitivity (Table 2). However, it should be noted that this would not exclude the possibility that other genes could become potential genotoxicity biomarkers.

Transcriptional expression of BC was induced by GTXs in NIH/3T3 cells, but not in L5178Y cells. We inferred that this discrepancy might be due to a lack of functional p53 in L5178Y cells [34]. A search for transcriptional factor binding sites revealed that GLN LTR has a putative p53 binding site (Fig. S7A). In a preliminary study on the mechanisms of BC expression regulation in NIH/3T3 cells, we found that the wild-type LTR had great promoter/enhancer activity, even stronger than the positive control using luciferase reporter gene assay (Fig. S7B). However, when the putative p53 binding site was deleted, the promoter/enhancer activity dropped significantly (Fig. S7B). To address the role of p53 in BC induction, we examined the effects of treatment with pifithrin-alpha (PFT-α), a widely used p53 inhibitor [35], on BC expression in NIH/3T3 cells. Consistent with our expectations, treatment with PFT-α decreased ETOP or MMS induced BC expression (Fig. S8). These results indicated that p53 might play a role in BC induction following GTXs treatment.

There are many genotoxicity mechanisms and the model compounds selected in this work could not be expected to cover all of them. More model compounds with diverse genotoxicity mechanisms should be tested to further examine the sensitivity and specificity of BC expression. An interesting finding in this study was that BC correlated with DNA damage but not chromosomal lesion, indicating that BC might not be appropriate in aneugen genotoxicity prediction. Given both the *in vivo* (Fig. 1 and Table 2) and *in vitro* data (Fig. 3), BC has the intrinsic ability to become a sensitive biomarker of DNA damage. Although BC induction was not obvious in response to colchicine and paclitaxel treatment, this would not compromise the sensitivity of BC as a potential biomarker of DNA damage, since colchicine and paclitaxel mainly target microtubules rather than DNA. Additionally, it should be noted that compounds that require S9 activation were excluded from the *in vitro* study, in order to avoid potential S9 effects on BC expression. It would be of great interest to examine the effects of indirect genotoxic chemicals on BC expression in future studies. Moreover, we are currently developing a GLN LTR driven luciferase reporter gene system in NIH/3T3 cells, to facilitate toxicity screening in early drug development [36], [37], [38], [39].

The GLN family was recently reported to be potentially active in the mouse genome [21], thus uncovering its cellular functions would be of great significance. To the best of our knowledge, this is the first report showing the involvement of a GLN family member in cell growth regulation, thus providing basic information towards a deeper understanding of its biological roles. Several studies have reported the mitogenic effects of exogenous retroviruses (XRVs) on certain cell lines [40], [41]. One suggested mechanism was the interaction of glycoproteins encoded by retroviral *env* genes with cellular cytokine receptors, such as interleukin-2 receptor or erythropoietin receptor. One study also suggested a role for *env* of mink cell focus-forming (MCF) ERV in regulating pluripotent hemopoietic progenitor proliferation. Given previous reports that GLN was potentially active and might have an extracellular life cycle similar to XRVs, it would be of great interest to study whether GLN regulates cell growth through similar mechanisms. Such studies will not only assist in a deeper understanding of its biological function, but also provide theoretical support for its application in genotoxicity prediction.

In summary, this is the first study to investigate the relationship between ERVs and genotoxicity. We showed that expression level of BC005512, a member from GLN family of murine ERV, was elevated by multiple GTXs both *in vivo* and *in vitro*, and correlated with the extent of DNA damage. It is thus possible that BC might be useful as a potential molecular biomarker for assessment of DNA damaging effects. Additionally, we first demonstrated that BC was involved in cell growth regulation. This contributes to a deeper understanding of the biological functions of BC005512 and GLN, and provides fundamental support for their application in genotoxicity predictions.

## Materials and Methods

### Ethics Statement

Animal-use protocols were approved by the Institutional Animal Care and Use Committee of the Shanghai Institute of Materia Medica (Shanghai, China) with IACUC No. 2010-10-RJ-05.

### Animal treatment in microarray study

Two-month-old male B6C3F1 mice were given a single intraperitoneal injection of model compounds or solvent control (corn oil or saline) as listed in Table 1. *O*-AAT, DBP, DMBA and DEHP were dissolved in corn oil while other chemicals were dissolved in saline. Mice were sacrificed at 4 h, 20 h, 14 days or 28 days after administration and liver samples were collected immediately. Animal administration was the same in the quantitative PCR confirmation experiment except that B6C3F1 mice were substituted with C57BL/6 mice (supplied by Shanghai Slac Laboratory Animal, Shanghai, China). DEHP and EtOH were purchased from Sinopharm Chemical Reagent (Shanghai, China), and other chemicals were from Sigma (St Louis, MO, USA).

### Microarray and data analysis

Five µg pooled total RNA of liver from 4 animals per group was used as starting material. For each time point of each compound, only one sample pooled from 4 animals was tested. cDNA synthesis, cRNA labeling, and cRNA fragmentation were conducted according to the manufacturer's instructions (Affymetrix Inc., Santa Clara, CA, USA). The hybridization mixture was hybridized to an Affymetrix Mouse Genome 430 2.0 array. Hybridized arrays were washed and stained, and fluorescence signals were detected using the Affymetrix GeneChip Scanner 3000. The image files were converted into expression data by the Microarray Suite Software (Affymetrix) and the data was imported into GeneSpring software (Silicon Genetics, Redwood City, CA, USA). Signal intensity was normalized by per-gene and per-chip. Ratio was calculated by normalizing treatment samples to solvent control samples. The procedure was in compliance to MIAME guidelines. The raw data has been deposited in GEO database (GSE33248).

We used a step-wise selection criterion (set different significance levels according to the intensity) in selecting differentially expressed genes. For each time point of each chemical, genes that met with either criterion listed in Table S1 were considered as up-regulated genes and the corresponding cells in the Table S3 were marked with pink. While genes that met with either criterion listed in Table S2 were considered as down-regulated genes and the corresponding cells in the Table S3 were marked with blue.

Those selected up-regulated genes were further analyzed by self-defined weight scoring based on several important parameters in identifying genotoxic stress responsive genes (including specificity, sensitivity, statistical P value, basal expression level, and coefficient of variation) Detailed scoring rules are described in supporting information (Text S1). Top 50 genes were further analyzed by hierarchical clustering (average linkage clustering) by using Gene Cluster and TreeView programs [42].

### Cell lines and culture

NIH/3T3, Hepa 1–6 and SV40 MES 13 cells were purchased from Type Culture Collection of Chinese Academy of Sciences (http://www.cellbank.org.cn/index.asp). L5178Y cells were kindly provided by Dr. M. Honma (NIHS, Japan) [43]. NIH/3T3 cells were cultured in Dulbecco's modified Eagle's medium (DMEM, Gibco BRL, Grand Island, NY, USA) supplemented with 10% calf serum (Sijiqing Biological Engineering Materials, Hangzhou, China). L5178Y cells were cultured in RPMI 1640 medium with 10% horse serum. Hepa 1–6 and SV40 MES 13 cells were cultured in DMEM with 10% fetal bovine serum (FBS, Gibco) for maintenance or in DMEM with 2.5% FBS for cell growth assay. All cells were maintained at 37°C in 5% CO_2_.

### Chemicals treatment in *in vitro* BC expression assay

Compounds that require metabolic activation were excluded to avoid potential effects of S9 fraction on BC expression. The seven selected genotoxins represent different mechanisms of action. NIH/3T3 or L5178Y cells were treated with indicated concentration of model compounds or irradiated with γ-ray by Gammacell 3000 Elan (MDS Nordion, Ottawa, ON, Canada) as listed in Table 3. After 4, 8, 12 and 24 h treatment or irradiation for NIH/3T3, 4 h for L5178Y, cells were harvested and subjected to RNA isolation and qPCR. MMC was purchase from Kyowa (Tokyo, Japan), ACN from Merck (Darmstadt, Germany), SUC from Sinopharm, and other chemicals were from Sigma.

### Quantitative real-time PCR (qPCR)

Total RNA of mouse liver, NIH/3T3, L5178Y, Hepa 1–6 or SV40 MES 13 cells was extracted by using the UNIQ-10 total RNA isolation kit (Sangon Biotech, Shanghai, China). The remnant genomic DNA in total RNA was digested by RNase-free DNase I (Fermentas, Burlington, Canada). DNase-digested RNA was further reverse transcribed into cDNA by using the PrimeScript RT reagent kit (TaKaRa, Otus, Shiga, Japan). DNase-digested RNA without reverse transcription was used as a negative control. qPCR was carried out by using the SYBR Premix Ex Taq (TaKaRa) with BC primers (forward: 5′-ATCACCCTGCATCCAGTTTAG -3′, reverse: 5′-TATTGCCGCTAGGTCTTCATT -3′) or GAPDH primers (forward: 5′-GGCTACACTGAGGACCAGGTT-3′, reverse: 5′-TGCTGTAGCCGTATTCATTGTC-3′). The qPCR conditions were as follows: 95°C, 10 s; (95°C, 5 s, 60°C, 34 s) 40 cycles, with a melting-curve process. Amplification process was performed on 7500 fast real-time PCR system (Applied Biosystems, Foster City, CA, USA) and data was analyzed by using 2^−ΔΔCt^ with the Sequence Detection Software.

### Alkaline comet assay

The alkaline comet assay was performed as previous described with slight modifications [28]. Briefly, NIH/3T3 cells were exposed to MMS for 8 h or to paclitaxel (Sigma) or colchicine (Sigma) for 24 h. Then cells were trypsinized, resuspended in PBS, mixed with 0.5% low-melting agarose and applied to glass slides pre-coated with 1% normal-melting agarose. Slides mounted with cells were immersed in cold lysing solution (2.5 M NaCl, 100 mM EDTA, 10 mM Tris, 1% Triton X-100, 10% DMSO, pH = 10; the last two compounds were added fresh) for 2.5 h. After lysis, slides were immersed in alkaline buffer (300 mM NaOH, 1 mM EDTA, pH = 13) for 20 min to allow DNA unwinding and DNA breakage at alkali-labile sites. Then electrophoresis was performed at 300 mA for 20 min. After electrophoresis, slides were neutralized in 0.4 M Tris-HCl, dipped in ethanol and air-dried. Cells were stained with SYBR Green and observed by a fluorescent microscope (Olympus BX 51). At lease 100 cells were pictured for each group, and olive tail moment (tail length×percentage of DNA in tail) was determined by image analysis software Komet 5.5 (Kinetic Imaging, Liverpool, UK).

### Micronucleus test

The micronucleus test was performed as described previously with some modifications [44] [31]. Briefly, NIH/3T3 cells were exposed to paclitaxel or colchicine for 24 h. Then the attached cells were trypsinized, incubated in 0.075 M KCl hypotonic solution, fixed in methanol-acetic acid (3∶1) and finally suspended in methanol containing 1% acetic acid. A drop of cell suspension was placed on a clean glass slide and air-dried. Cells were stained by mounting with acridine orange and immediately observed by a fluorescent microscope (Olympus BX 51). The micronucleus frequency was determined in 1000 total cells according to published criteria [44], and cells with irregular shape of nucleus were also counted as micronucleated cells. Cytochalasin B was not used since it has been reported that the alteration of two cytoskeletal elements, microtubules and microfilaments, concomitantly could influence the formation of micronucleated cells [45].

### Cell transfection

Transfection of siRNA into NIH/3T3, Hepa 1–6 or SV40 MES cells was conducted by using RNAiMAX (Invitrogen) with 50 pmol siRNA per 35 mm dish. BC siRNA-1 (siBC.1) targeting 5′-CAGGUACCUCUAACUAUUAdTdT-3′, BC siRNA-2 (siBC.2) targeting 5′-CCAGUUUAGAAGAAAGCUAdTdT-3′, nonsense siRNA targeting 5′- GCGACGAUCUGCCUAAGAUdTdT-3′, scrambled-siBC.1 targeting 5-GAUCGAUAACCCAUCUUUAdTdT-3 and scrambled-siBC.2 targeting 5-GCUAAUACUAGGCAAUGAAdTdT-3 were synthesized by GenePharma (Shanghai, China). All siRNAs were chemically modified with 2′-fluoro-dU and 2′-fluoro dC.

### Cell growth assay

At 24, 48, 72 or 96 h after siRNA transfection, NIH/3T3, Hepa 1–6 or SV40 MES cells were trypsinized and cell numbers were determined by using a Z1 Coulter counter (Beckman Coulter, Fullerton, CA, USA).

### EdU incorporation assay

EdU incorporation assay was performed by using Cell-Light EdU DNA imaging kit (RiboBio, Guangzhou, China) according to the manufacture's instructions. Briefly, at 48 h after siRNA transfection, NIH/3T3 cells were cultured in medium containing 50 µM EdU for 3.5 h. After EdU incorporation, cells were fixed in 4% paraformaldehyde and permeabilized by 0.5% Triton X-100. After a click reaction between Apollo dye and ethynyl group in EdU, EdU positive cells were visualized with a green light excitation (550 nm), while total cells were visualized by Hoechst 33342 staining with UV excitation. At least a total of 4000 cells were pictured for each group. EdU positive and total cells were counted by using software Image-Pro Plus (Media Cybernetics, Bethesda, MD, USA).

### Cell cycle analysis

At 48 h after siRNA transfection, NIH/3T3 cells were trypsinized, fixed in 70% ethanol, incubated with RNaseA, stained with propidium iodide (Sigma) and analyzed by a FACSCalibur (BD, Franklin Lakes, NJ, USA) instrument. Analysis of cell cycle phase distribution was performed by using the Modfit software (Verity Software House, Topsham, ME, USA).

### Statistical analysis

Data were analyzed by Student's unpaired *t*-test in Excel. Differences were considered significant at *P<0.05, **P<0.01. For BC expression study, the raw data was divided by that of control, generating the normalized BC expression (folds of control). For linear regression analysis, each dot in Fig. 4C represents the mean of data shown in Fig. 4A, 4D and 4G. The linear regression analysis was performed by using GraphPad Prism 5 (GraphPad Software, Inc., La Jolla, CA, USA).

## Supporting Information

Text S1 **Supporting materials and methods.**
(DOC)

Figure S1 **Probe sequence (1426936_at) in Affymetrix Mouse Genome 430 2.0 array.**
(TIF)

Figure S2 **Expression of BC was induced by GTXs in Hepa 1–6 cells.** Quantitative PCR data showing transcriptional expression of BC in Hepa 1–6 cells treated with CPT (3 µM), MMC (100 µM), ETOP (50 µM) or MMS (0.5 mM) for indicated times. Dose was 24 h IC_50_. Data were mean ± s.d. of three independent experiments.
(TIF)

Figure S3 **Effects on BC expression in L5178Y cells following MMS treatment at various time points.** L5178Y cells were treated with indicated concentrations of MMS. At various time points after MMS incubation, cells were harvested and expression levels of BC were analyzed by quantitative PCR. Data were mean ± s.d. of three independent experiments.
(TIF)

Figure S4 **Specific effects of BC siRNAs on cell growth and cell cycle progression.** Expression level of BC (**A**), cell number (**B**) and cell cycle phase distribution (**C**) in NIH/3T3 cells transfected with indicated siRNAs at 48 h after transfection. Data were mean ± s.d. of at least three independent experiments performed in triplicate. Values shown on top of bars are the *P* values *vs* corresponding scrambled siRNA.
(TIF)

Figure S5 **Representative western blot results showing protein level knock-down efficiency and specificity of siBC.2.** NIH/3T3 cells were co-transfected with wild-type or mutant myc tagged BC clone with nonsense siRNA or siBC.2. Cell lysates were collected at 24 h after co-transfection. β-Actin served as a loading control. Primary antibody against Myc tag was from Cell Signaling (Danvers, MA, USA).
(TIF)

Figure S6 **Flow cytometric analysis of apoptosis in NIH/3T3 cells.** NIH/3T3 cells were transfected with indicated siRNAs. At 48 or 96 h after transfection, apoptosis was determined using Annexin V-FITC Apoptosis Detection Kit (BD Pharmingen) and a FACSCalibur (BD Pharmingen) instrument. The lower right quadrant cells indicate early apoptotic cells, while the upper right quadrant cells indicate late-apoptotic or dead cells.
(TIF)

Figure S7 **The promoter/enhancer activity of wild-type and p53 binding site deleted LTRs of GLN.** (**A**) LTR sequence of GLN. The sequence of the putative p53 binding site is shown in bold and is consistent with the consensus p53 sequence (5′-RRRCWWGYYY-3′, R = purine, Y = pyrimidine, W = A or T). This putative p53 binding site was composed of two half-site RRRCWWGYYY with no spacers. Right arrows indicate RRRCW and left arrows indicate WGYYY. (**B**) Luciferase assay of the GLN LTRs in NIH/3T3 cells. “Basic” and “Control” represents negative and positive control respectively. LTR-D represents LTR with the putative p53 binding site deletion. LTR-WT represents wild-type LTR. Data were mean ± s.d. of at least three independent experiments.
(TIF)

Figure S8 **Effects of pifithrin-alpha (PFT-α) treatment on GTX-induced BC expression in NIH/3T3 cells.** NIH/3T3 cells were treated with ETOP or MMS in combination with indicated concentrations of PFT-α. Cells were harvested after 24 h and BC expression was analyzed by quantitative PCR. Data were mean ± s.d. of three independent experiments. *P<0.05, **P<0.01 *vs* PFT-α 0 µM.
(TIF)

Table S1 **Step-wise criterion in selecting up-regulated genes.**
(DOC)

Table S2 **Step-wise criterion in selecting down-regulated genes.**
(DOC)

Table S3 **Raw data and detailed scoring rule of up-regulated genes.**
(XLS)
