# Supplementary material for: Identification of BC005512 as a DNA Damage Responsive Murine Endogenous Retrovirus of GLN Family Involved in Cell Growth Regulation
Source: PLoS One. 2012 Apr 13;7(4):e35010. doi: 10.1371/journal.pone.0035010 (PMC3325921; doi:10.1371/journal.pone.0035010)
Supplement: Text S1 — Supporting materials and methods. (DOC) [file pone.0035010.s001.doc]

**Supporting Materials and Methods**

**1. Differentially expressed gene selection and weight scoring rules in the microarray study**

We used a step-wise selection criterion (set different significance levels according to the intensity) in selecting differentially expressed genes. Details were described in Table S1 and S2. Those selected up-regulated genes were further analyzed by self-defined weight scoring.

**Specificity**

Specificity= (number of total pink cells in GTXs) / (number of total pink cells in GTXs and NGTXs)

Scoring rule:

| **Specificity** | **Score** |
| --- | --- |
| =1 | 5 |
| 0.9-1 | 4 |
| 0.8-0.9 | 3 |
| 0.7-0.8 | 2 |
| 0.6-0.7 | 1 |
| <0.6 | 0 |

**Ave ratio**

Ave ratio=average of ratios of all pink cells in GTXs

Scoring rule:

| **Ave ratio** | **Score** |
| --- | --- |
| >10 | 5 |
| 6-10 | 4 |
| 4-6 | 3 |
| 2-4 | 2 |
| <2 | 0 |

**Positive condition**

Positive condition was calculated as number of total pink cells in GTXs. Since DEN was duplicated, each pink cell was considered 0.5

Scoring rule:

| **Positive condition** | **Score** |
| --- | --- |
| >10 | 5 |
| 8-10 | 4 |
| 6-8 | 3 |
| 4-6 | 2 |
| 2-4 | 1 |
| 0-2 | 0 |

**Positive chemical**

Positive chemicals =number of GTXs with at least one pink cell

Scoring rule:

| **Positive Chemical** | **Score** |
| --- | --- |
| ≥6 | 5 |
| 5 | 4 |
| 4 | 2 |
| ≤3 | 0 |

**P Value**

P value was calculated by *t* test of signal intensity between GTXs and NGTXs in Genespring software.

Scoring rule:

| **P value** | **Score** |
| --- | --- |
| <0.15 | 5 |
| 0.15-0.25 | 4 |
| 0.25-0.3 | 3 |
| 0.3-0.35 | 2 |
| 0.35-0.4 | 1 |
| >0.4 | 0 |

**Basal**

Basal represents basal expression level, was calculated Log_10_ value of signal intensity of control animals.

Scoring rule:

| **Basal** | **Score** |
| --- | --- |
| >3 | 5 |
| 2.5-3 | 4 |
| 2-2.5 | 3 |
| 1.5-2 | 2 |
| 1-1.5 | 1 |
| <1 | 0 |

**Reverse change**

Reverse change reflects opposite change of gene expression in different treatment groups. Reverse change=number of blue cells in NGTXs - number of blue cells in GTXs

**Scoring rule:**

| **Reverse change** | **Score** |
| --- | --- |
| ≥2 | 5 |
| =1.5 | 4 |
| =1 | 3 |
| =0 | 2 |
| =-1 | 1 |
| ≤-2 | 0 |

**CV%**

CV% was calculated as CV%=100×SD/MEAN% based on the signal intensity of all control animals.

Scoring rule:

| **CV (%)** | **Score** |
| --- | --- |
| 0-20 | 5 |
| 20-40 | 4 |
| 40-60 | 3 |
| 60-80 | 2 |
| 80-100 | 1 |
| >100 | 0 |

**Total score**

Total score=Score of 2×Specificity + 1× (Ave ratio + Positive condition + Positive chemical + P value) + 0.5× (Basal + Reverse change + CV %)

**2. Constructs**

A BC partial sequence encompassing the siBC.2 region from NIH/3T3 cells was cloned into pcDNA3.1-Myc-His-B with primers 5’-CGGGATCCCGCCACCATGCCTGTCCTCGGGTCCAC-3’ (forward) and 5’-CCCAAGCTTCCATCTTTCAGTTTCTGCATAG-3’ (reverse) to generate a wild-type myc-tagged BC construct. The mutant construct was generated by introducing two point silent mutations in the siBC.2 target sequence by using the site-directed mutagenesis kit (SBS Bio, Beijing, China). PCR reaction was performed with primers 5’-CCCTGCATCCAGT(C)T(G)GAAGAAAGCTATGCTGGC-3’ (forward) and 5’-GCCAGCATAGCTTTCTTC(C)A(G)ACTGGATGCAGGG-3’ (reverse). Primer sequences underlined correspond to siBC.2, and letters with parentheses indicate point mutations. All PCR primers were synthesized by SBS Bio (Beijing, China).

**3. Co-transfection**

Co-transfection of plasmid (wild-type or mutant myc-tagged BC clone) and siRNA (BC or nonsense siRNA) into NIH/3T3 cells was performed by using Lipofectamine 2000 (Invitrogen) with 2 μg plasmid and 100 pmol siRNA per 35mm dish. At 24 h after transfection, cells were lysed in 1×SDS buffer and subjected to western blot for the analysis of BC-Myc fusion protein.

**4. Western blot**

Protein quantitation was performed by using BCA protein assay kit (Pierce, Rockford, IL, USA). 30 μg total protein were separated on SDS-PAGE, transferred to nitrocellulose membrane (Amersham, Buckinghamshire, UK), hybridized with primary antibodies and secondary antibodies conjugated with horseradish peroxidase. Protein bands were visualized by the ECL detection system (Amersham). Their densities were determined by ImageQuant 5.2 software (Amersham). Primary antibody against myc or β-Actin was from Santa Cruz (Santa Cruz, CA, USA), and secondary antibody from DingGuo (Beijing, China).

**5. Luciferase reporter gene assay**

The wild-type LTR and p53 binding site deficient LTR were synthesized by Integrated Biotech Solutions Co. (Shanghai, China) and cloned into pGL3-basic (Promega). NIH/3T3 cells were co-transfected with pGL3-basic (negative control) / pGL3-control (containing SV40 promoter and enhancer sequences, positive control, Promega) / pGL3-LTR-D-basic / pGL3-LTR-WT-basic and pSV-β-galactosidase (internal control, Promega) by using Fugene 6 (Roche), with 1 μg luciferase vector and 1 μg pSV-β-galactosidase per 35 mm dish. At 24 h after transfection, cell lysate was collected. Luciferase assay was performed by using luciferase assay system (Promega) as manufacture’s instructions, and luminescence was measured in a Synergy 4 multi-mode microplate reader (Biotek, Winooski, VT, USA). β-gal activity was determined by using o-Nitrophenyl β-D-galactopyranoside (ONPG) as a substrate and absorbance was measured at 420 nm. The output luciferase activity data was corrected by β-gal activity and then normalized to the pGL3-basic group.
