# Supplementary figures and images for: Identification of BC005512 as a DNA Damage Responsive Murine Endogenous Retrovirus of GLN Family Involved in Cell Growth Regulation
Source: PLoS One. 2012 Apr 13;7(4):e35010. doi: 10.1371/journal.pone.0035010 (PMC3325921; doi:10.1371/journal.pone.0035010)

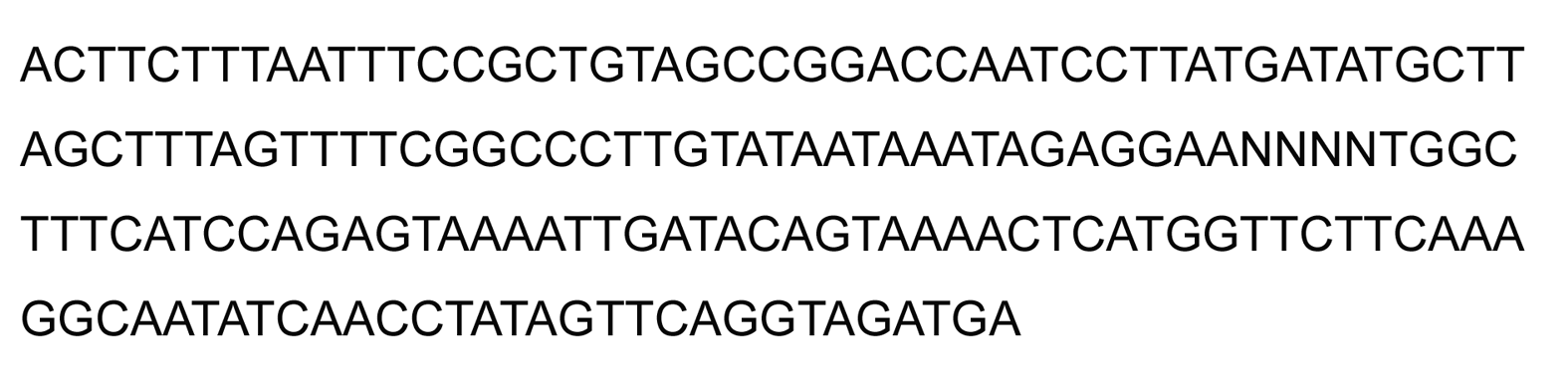

Supplement: Figure S1 — Probe sequence (1426936_at) in Affymetrix Mouse Genome 430 2.0 array. (TIF) [file pone.0035010.s002.tif]

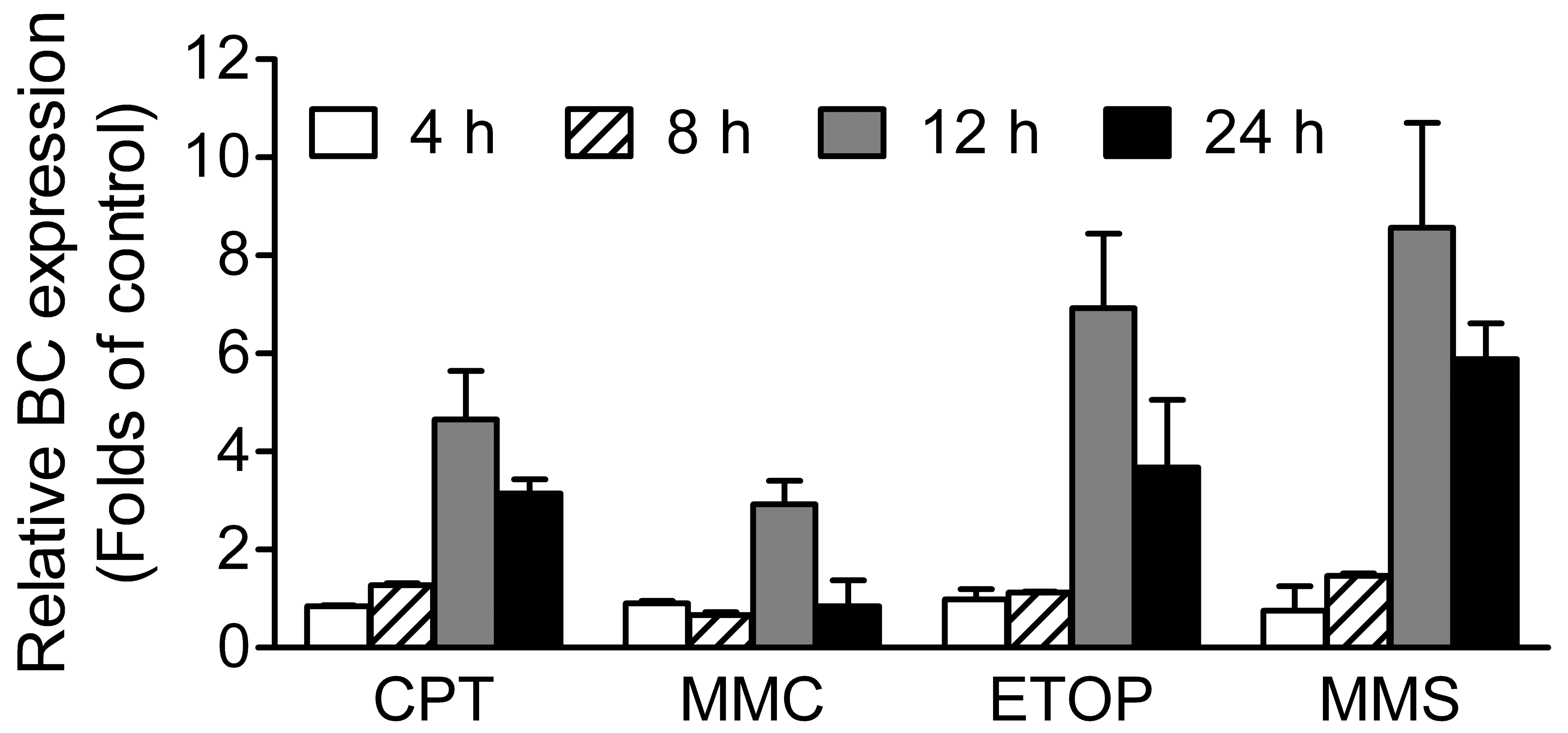

Supplement: Figure S2 — Expression of BC was induced by GTXs in Hepa 1–6 cells. Quantitative PCR data showing transcriptional expression of BC in Hepa 1–6 cells treated with CPT (3 µM), MMC (100 µM), ETOP (50 µM) or MMS (0.5 mM) for indicated times. Dose was 24 h IC50. Data were mean ± s.d. of three independent experiments. (TIF) [file pone.0035010.s003.tif]

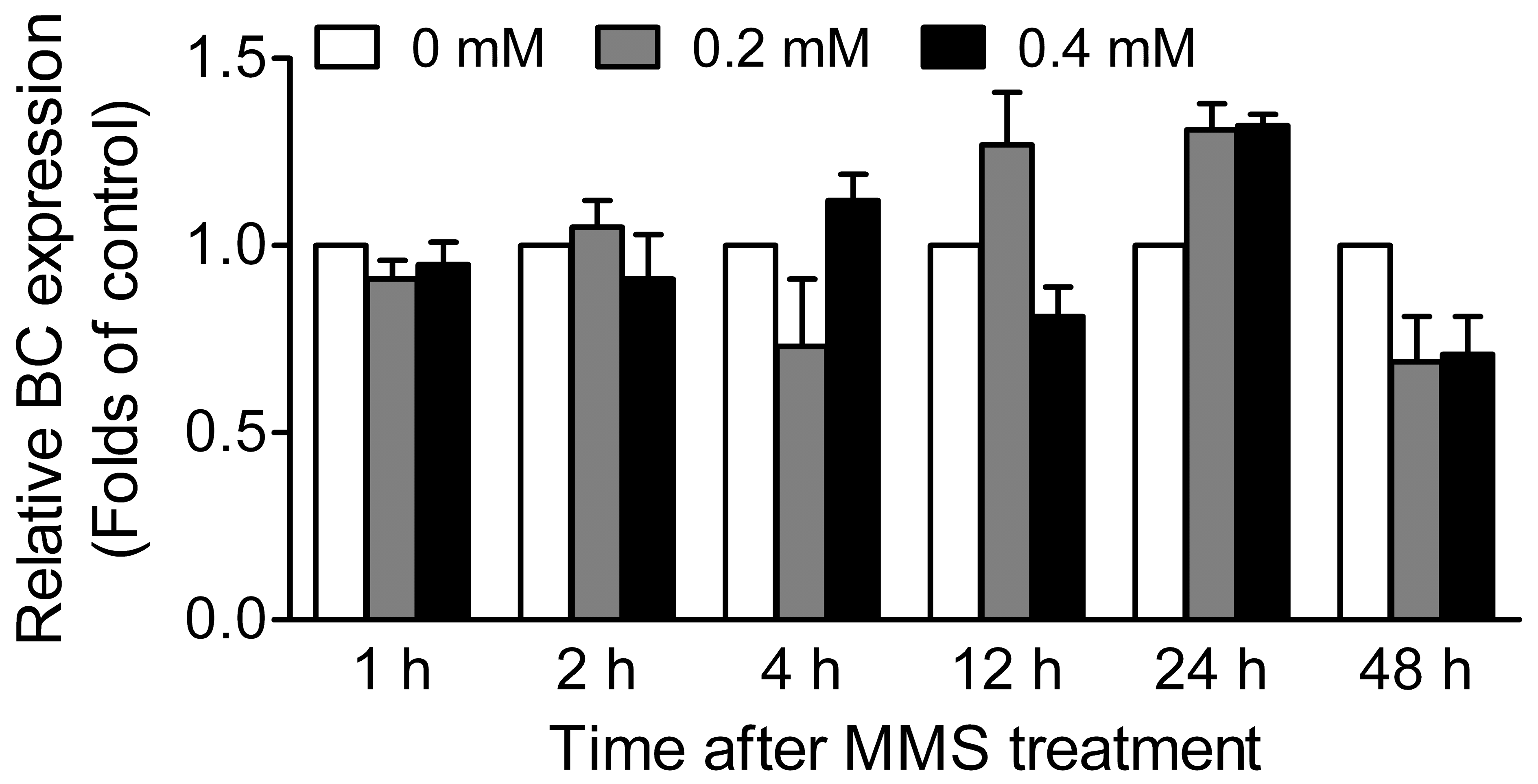

Supplement: Figure S3 — Effects on BC expression in L5178Y cells following MMS treatment at various time points. L5178Y cells were treated with indicated concentrations of MMS. At various time points after MMS incubation, cells were harvested and expression levels of BC were analyzed by quantitative PCR. Data were mean ± s.d. of three independent experiments. (TIF) [file pone.0035010.s004.tif]

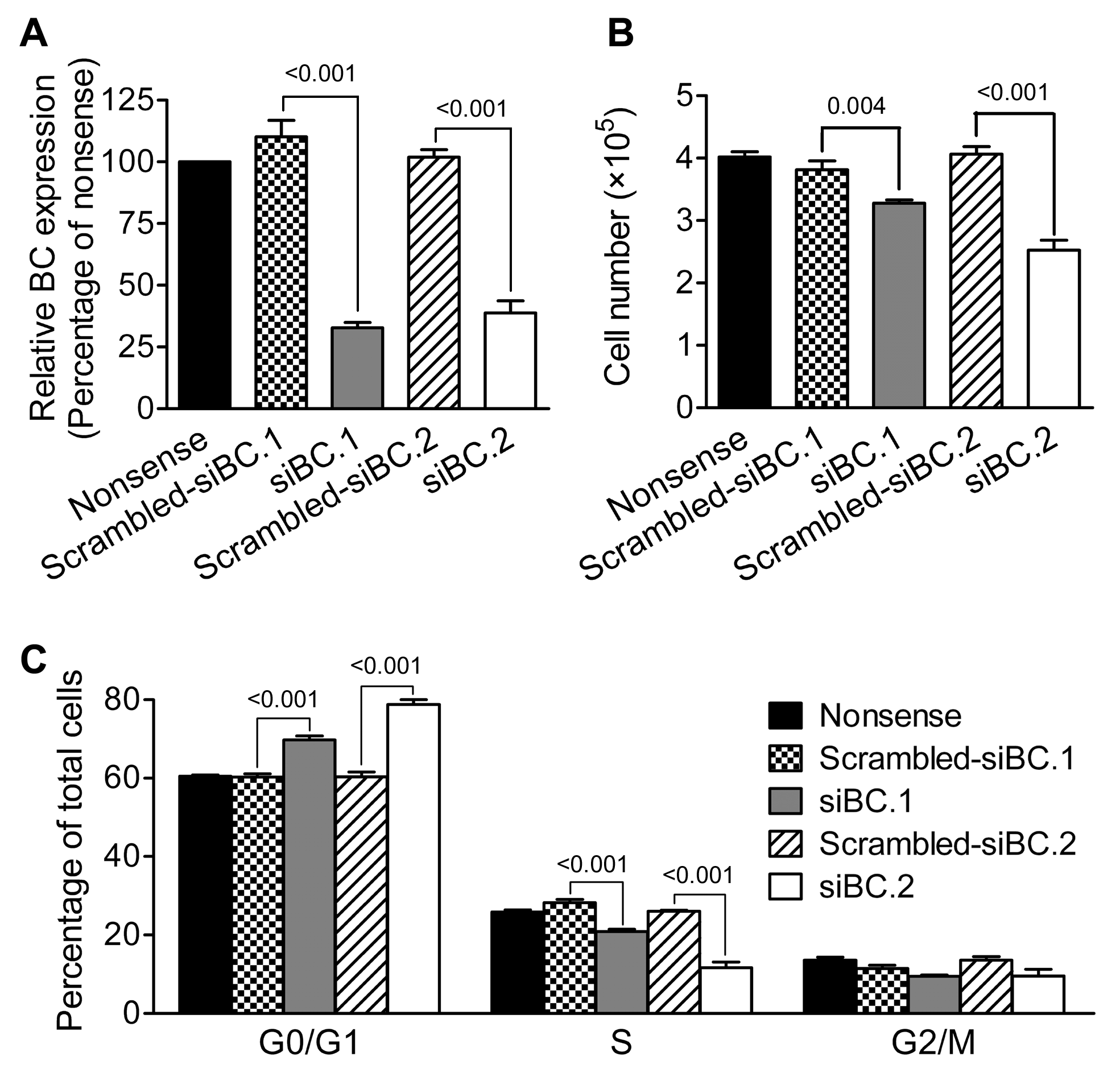

Supplement: Figure S4 — Specific effects of BC siRNAs on cell growth and cell cycle progression. Expression level of BC (A), cell number (B) and cell cycle phase distribution (C) in NIH/3T3 cells transfected with indicated siRNAs at 48 h after transfection. Data were mean ± s.d. of at least three independent experiments performed in triplicate. Values shown on top of bars are the P values vs corresponding scrambled siRNA. (TIF) [file pone.0035010.s005.tif]

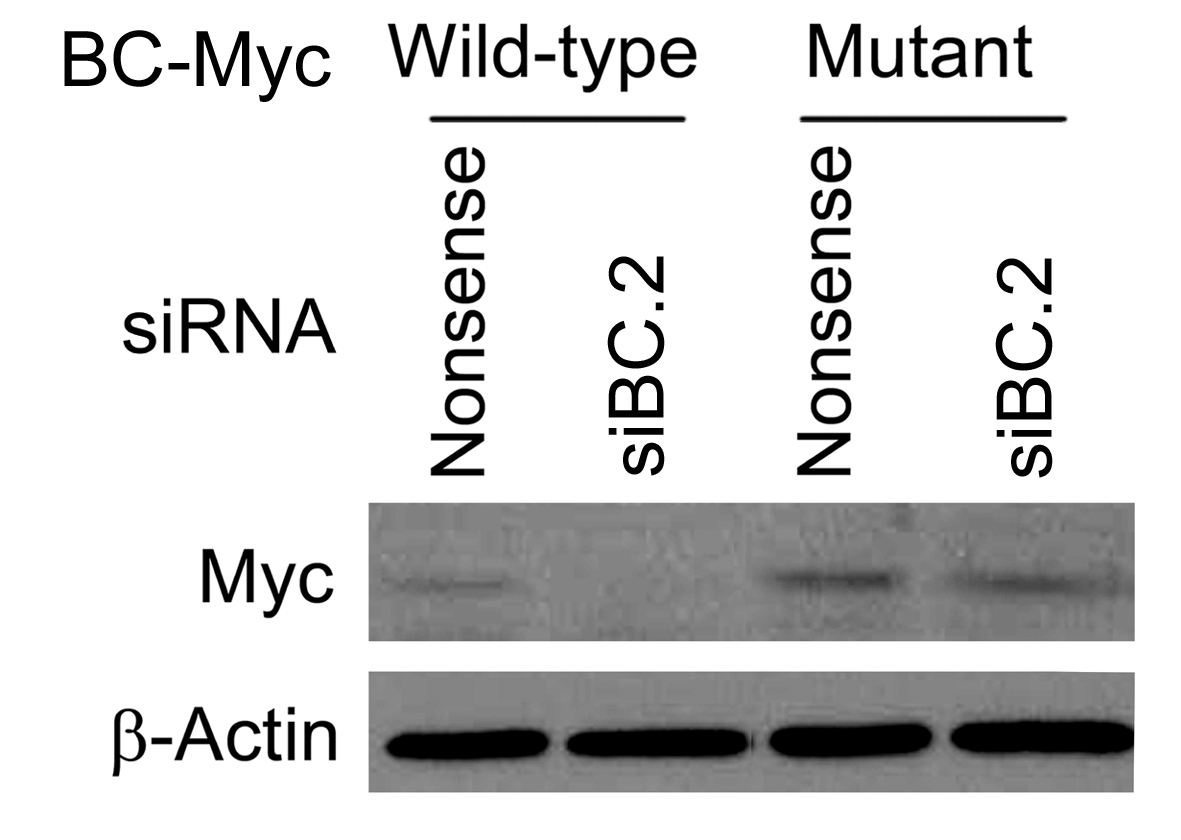

Supplement: Figure S5 — Representative western blot results showing protein level knock-down efficiency and specificity of siBC.2. NIH/3T3 cells were co-transfected with wild-type or mutant myc tagged BC clone with nonsense siRNA or siBC.2. Cell lysates were collected at 24 h after co-transfection. β-Actin served as a loading control. Primary antibody against Myc tag was from Cell Signaling (Danvers, MA, USA). (TIF) [file pone.0035010.s006.tif]

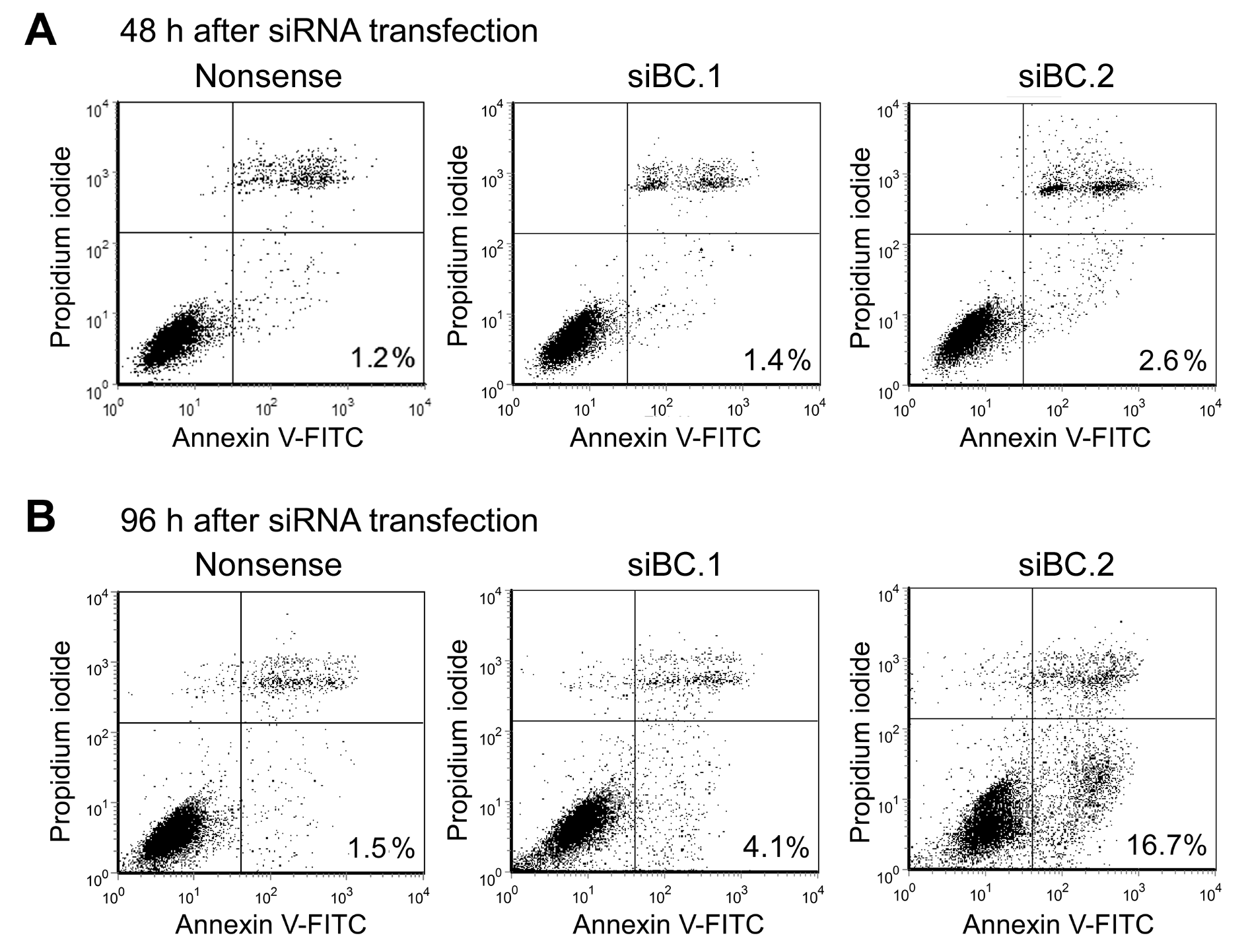

Supplement: Figure S6 — Flow cytometric analysis of apoptosis in NIH/3T3 cells. NIH/3T3 cells were transfected with indicated siRNAs. At 48 or 96 h after transfection, apoptosis was determined using Annexin V-FITC Apoptosis Detection Kit (BD Pharmingen) and a FACSCalibur (BD Pharmingen) instrument. The lower right quadrant cells indicate early apoptotic cells, while the upper right quadrant cells indicate late-apoptotic or dead cells. (TIF) [file pone.0035010.s007.tif]

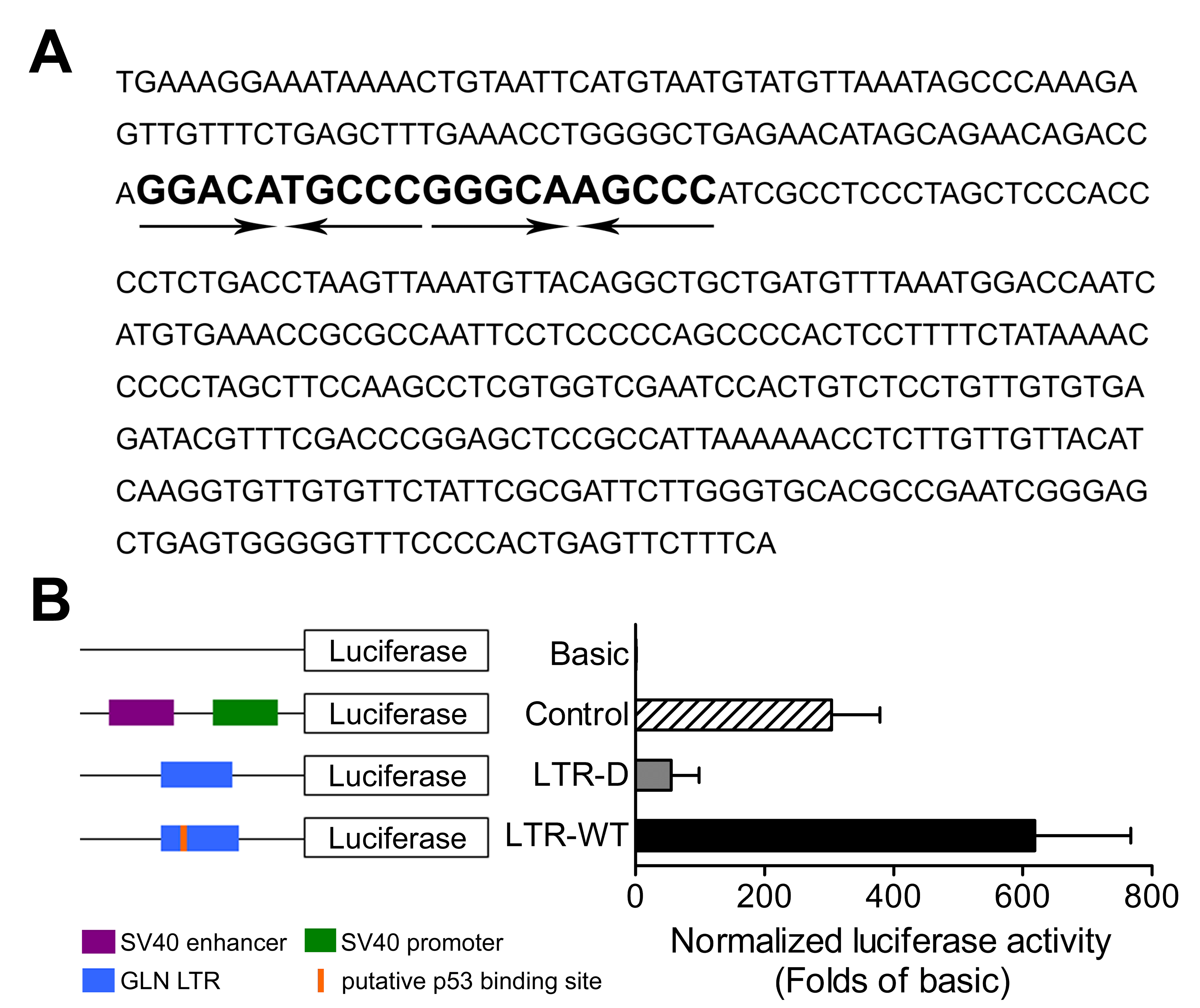

Supplement: Figure S7 — The promoter/enhancer activity of wild-type and p53 binding site deleted LTRs of GLN. (A) LTR sequence of GLN. The sequence of the putative p53 binding site is shown in bold and is consistent with the consensus p53 sequence (5′-RRRCWWGYYY-3′, R = purine, Y = pyrimidine, W = A or T). This putative p53 binding site was composed of two half-site RRRCWWGYYY with no spacers. Right arrows indicate RRRCW and left arrows indicate WGYYY. (B) Luciferase assay of the GLN LTRs in NIH/3T3 cells. “Basic” and “Control” represents negative and positive control respectively. LTR-D represents LTR with the putative p53 binding site deletion. LTR-WT represents wild-type LTR. Data were mean ± s.d. of at least three independent experiments. (TIF) [file pone.0035010.s008.tif]

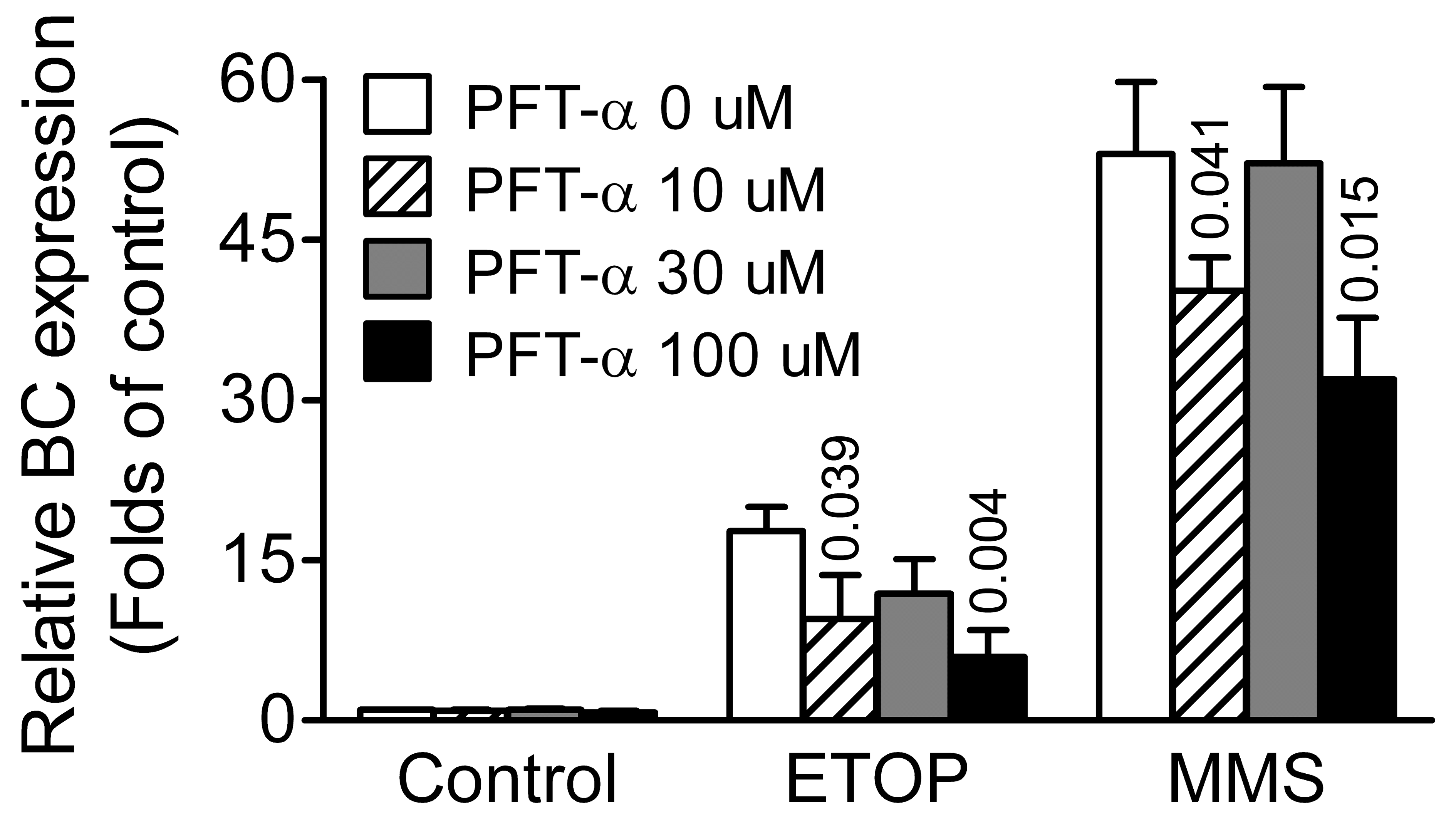

Supplement: Figure S8 — Effects of pifithrin-alpha (PFT-α) treatment on GTX-induced BC expression in NIH/3T3 cells. NIH/3T3 cells were treated with ETOP or MMS in combination with indicated concentrations of PFT-α. Cells were harvested after 24 h and BC expression was analyzed by quantitative PCR. Data were mean ± s.d. of three independent experiments. *P<0.05, **P<0.01 vs PFT-α 0 µM. (TIF) [file pone.0035010.s009.tif]
