# Supplementary material for: Identification of BC005512 as a DNA Damage Responsive Murine Endogenous Retrovirus of GLN Family Involved in Cell Growth Regulation
Source: PLoS One. 2012 Apr 13;7(4):e35010. doi: 10.1371/journal.pone.0035010 (PMC3325921; doi:10.1371/journal.pone.0035010)
Supplement: Table S1 — Step-wise criterion in selecting up-regulated genes. (DOC) [file pone.0035010.s010.doc]

**Table S1. Step-wise criterion in selecting up-regulated genes**

| **Treatment’s intensity** | **Ratio (Treatment / control)** | **Treatment’s Call** |
| --- | --- | --- |
| >1000 | >1.75 | Presence |
| 500-1000 | >2.0 | Presence |
| 100-500 | >2.25 | Presence |
| 50-100 | >2.5 | Presence |
| 10-50 | >3.5 | Presence |
